# Impact of Metabolically Healthy Obesity on Cardiovascular Outcomes in Older Adults with HFpEF: Insights from a Nationwide Sample

**DOI:** 10.3390/jcm14155495

**Published:** 2025-08-04

**Authors:** Adil Sarvar Mohammed, Hafeezuddin Ahmed, Sachin Singh, Cyrus Mutinda Munguti, Lakshmi Subramanian, Sashwath Srikanth, Lakshmi Sai Meghana Kodali, Maya Asami Takagi, Umera Yasmeen, Hassaan Imtiaz, Akhil Jain, Saad Chaudhry, Rupak Desai

**Affiliations:** 1 Department of Internal Medicine, Central Michigan University, Saginaw, MI 48602, USA; singh23s@cmich.edu; 2 Department of Internal Medicine, DMC Sinai Grace–Detroit Medical Center, Detroit, MI 48235, USA; drhafeez01@gmail.com; 3 Department of Cardiovascular Medicine, University of Kansas Medical Center, Kansas City, KS 66160, USA; cmunguti@kumc.edu; 4 Department of Cardiovascular Medicine, University of Tennessee Health Science Center, Chattanooga, TN 37403, USA; ramya.subramanian20@gmail.com; 5 Department of Cardiovascular Medicine, Baylor Medical Center, The Heart Hospital, Plano, TX 75093, USA; sashwath17@gmail.com; 6 Department of Public Health & Health Sciences, University of Michigan-Flint, Flint, MI 48502, USA; klsmeghu@umich.edu; 7 Department of Internal Medicine, University of California Davis, Sacramento, CA 95817, USA; atakagi@ucdavis.edu; 8 Department of Internal Medicine, Mamata Medical College, Khammam 507002, India; umera.uy@gmail.com; 9 Department of Internal Medicine, McLaren Health, Bay City, MI 48708, USA; hassaan.imtiaz214@gmail.com; 10 Department of Hematology and Oncology, University of Iowa Hospital and Clinics, Iowa City, IA 52242, USA; akhijain@uiowa.edu; 11 Department of Geriatrics, Central Michigan University, Saginaw, MI 48602, USA; chaud6s@cmich.edu; 12 Independent Outcomes Researcher, Atlanta, GA 30033, USA

**Keywords:** metabolically healthy obesity, HFpEF, dysrhythmia, MACCE, all-cause mortality, acute myocardial infarction

## Abstract

**Background:** Clinical outcomes among older adults hospitalized with heart failure with preserved ejection fraction (HFpEF) in the setting of metabolically healthy obesity (MHO) remain insufficiently explored. This study aimed to evaluate whether MHO status is associated with different rates of major adverse cardiac and cerebrovascular events (MACCEs) during HFpEF-related hospitalizations compared to patients without MHO. **Methods:** Data from the 2019 National Inpatient Sample (NIS) database was analyzed using relevant ICD-10 codes to identify HFpEF admissions in older adults. Propensity score matching (1:1) was applied to generate balanced cohorts of patients with and without MHO. Multivariable adjustments were performed to assess primary outcomes, including MACCEs, all-cause mortality (ACM), acute myocardial infarction (AMI), dysrhythmia, cardiac arrest (CA), and stroke. Statistical significance was set at *p* < 0.05. **Results:** Each MHO cohort included 22,405 patients with a median age of 75 years. The MHO+ group demonstrated a significantly higher risk of dysrhythmia (OR 1.32, 95% CI 1.21–1.43, *p* < 0.001). Interestingly, an “obesity paradox” was observed, as the MHO+ cohort had lower odds of MACCEs (OR 0.70, 95% CI 0.61–0.81, *p* < 0.001), ACM (OR 0.66, 95% CI 0.54–0.82, *p* < 0.001), and AMI (OR 0.71, 95% CI 0.59–0.86, *p* = 0.001) compared to MHO−. No significant differences were found for CA or stroke between the groups. **Conclusions:** Although the MHO+ group had an elevated risk of dysrhythmia, they exhibited more favorable outcomes in terms of MACCEs, ACM, and AMI—supporting the concept of an “obesity paradox.” Further research is needed to better understand the role of MHO as a comorbid condition in patients with HFpEF.

## 1. Introduction

Obesity exhibits a significant correlation with cardiovascular disease, functioning as an independent risk factor for increased morbidity and mortality [1]. The contemporary surge in global as well as national prevalence of obesity has prompted increased scrutiny of its ramifications on cardiovascular health. There is a strong association between obesity and heart failure (HF), notably with heart failure with preserved ejection fraction (HFpEF) [2]. Myocardial dysfunction in obesity secondary to an array of metabolic, endocrine, paracrine, neurohormonal mechanisms, and induced lipotoxicity increases the risk associated with HFpEF [3]. Despite this heightened risk associated with obesity, there exists a subgroup of individuals with obesity in the absence of diabetes, hypertension, and hyperlipidemia—popularly classified as metabolically healthy obesity (MHO) [4]—that challenges conventional expectations in cardiovascular disease outcomes. MHO introduces a paradoxical dimension to the relationship between obesity and cardiovascular outcomes. There is a scarcity of data on the outcomes of MHO on older adult patients hospitalized with HFpEF, particularly on outcomes like major adverse cardiovascular and cerebrovascular events (MACCEs) as compared to patients without MHO. Our study sheds light on the effects of MHO on MACCE outcomes in this cohort.

## 2. Methods

### 2.1. Data Source

We conducted the study using the National Inpatient Sample (NIS) database (2019) of the Healthcare Cost and Utilization Project (HCUP) sponsored by the Agency for Healthcare Research and Quality (AHRQ) [5]. The NIS is the most extensive publicly accessible all-payer inpatient healthcare dataset in the United States. It encompasses discharge data from approximately 20% of the hospitals across more than 48 states, covering an average of 7 million unweighted discharges annually. The data approximates to over 35 million weighted discharges nationwide. The NIS includes one primary diagnosis along with up to 34 secondary discharge diagnoses for each inpatient admission. Institutional Review Board (IRB) approval was not required as NIS data is deidentified.

### 2.2. Study Population

We identified adult hospitalizations among patients aged 65 years and older using the NIS 2019 dataset. Patients with HFpEF were selected using ICD-10-CM codes under the I50.3x classification, consistent with the Clinical Classifications Software Refined (CCSR) methodology. To refine the cohort and minimize confounding, we excluded patients with heart failure with reduced ejection fraction (HFrEF) using ICD-10-CM codes I50.2x, as well as those with a diagnosis of hypertension, diabetes mellitus, or hyperlipidemia, based on relevant ICD-10-CM codes. In line with widely accepted definitions, we defined MHO as the presence of obesity in the absence of common metabolic comorbidities such as hypertension, hyperlipidemia, and diabetes [6]. From this metabolically healthy HFpEF cohort, we stratified patients into two groups based on obesity status. Obesity and other comorbidities were defined using ICD-10 codes under Elixhauser comorbidity index [BMI cut off > 30]. Individuals with obesity were identified using ICD-10-CM codes (listed in Supplement Table S1), resulting in two cohorts: MHO and metabolically healthy non-obese (MHNO) patients. These final groups represented older adult patients (≥65 years) with HFpEF and no metabolic comorbidities other than obesity. All subsequent analyses, including demographic characteristics, comorbidities, and cardiovascular outcomes, were performed on these two cohorts.

### 2.3. Statistical Analysis

Baseline demographics and hospital characteristics between the MHO and MHNO groups were compared using the Pearson chi-square test for categorical variables and the Mann–Whitney U test for continuous variables, given their non-normal distribution. Categorical variables were reported as percentages, and continuous variables were expressed as mean ± standard deviation (SD). To minimize confounding and ensure balanced comparison, we conducted 1:1 propensity score matching using a nearest-neighbor algorithm without replacement. The covariates used for matching included age, sex, ethnicity, income quartile, primary payer, admission type, hospital bed size, teaching status, and region. Covariate balance between matched groups was assessed using standardized mean differences (SMDs), with a threshold of <0.1 considered indicative of adequate balance. Post-matching, multivariable logistic regression was used to calculate adjusted odds ratios (aORs) and 95% confidence intervals (CIs) for key in-hospital outcomes. A two-tailed *p* value of <0.05 was considered statistically significant. All statistical analyses were performed using IBM SPSS Statistics version 25.0 (IBM Corp., Armonk, NY, USA).

## 3. Results

**Figure 1:**
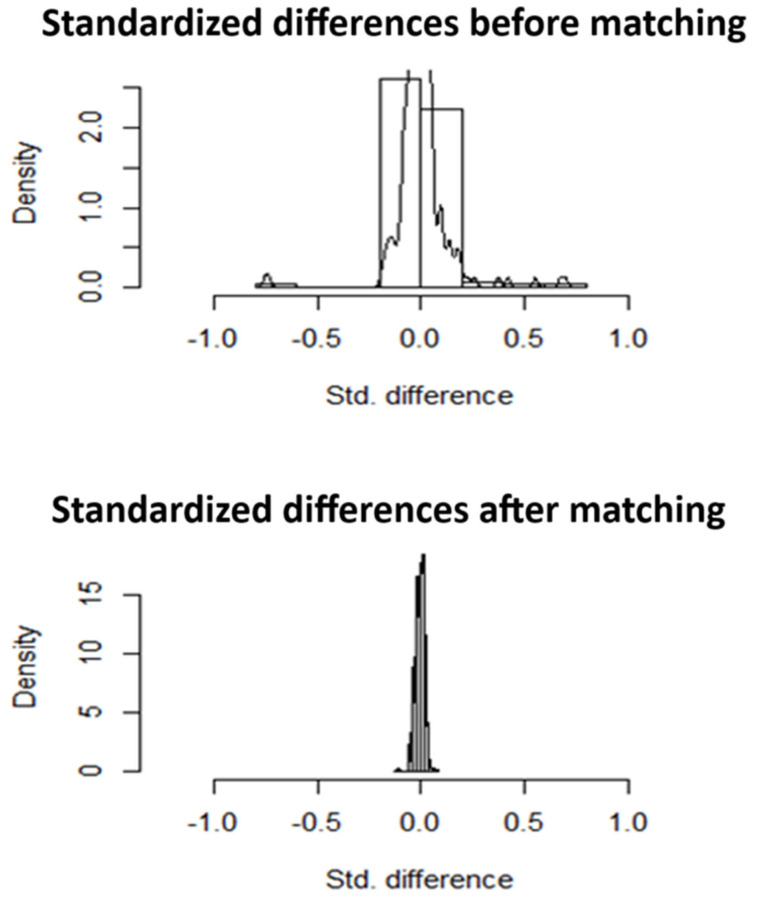
Standardized differences before and after propensity matching.

Out of 151,000 total admissions among metabolically healthy patients with HFpEF and age greater than 65, 15.6% (23,565) had MHO. After propensity matching (Figure 1), there were 22,405 patients with and without MHO. The median age at admission was 75 years, and in both groups, a higher proportion of females of Caucasian descent was seen. The primary expected payer was Medicare for most of the patients.

MHO patients exhibited higher rates of baseline comorbidities, with a higher prevalence of chronic obstructive pulmonary disease (COPD) [51.6% vs. 45.6%, *p* < 0.001], hypothyroidism [23.1% vs. 18.9%, *p* < 0.001], prior venous thromboembolism (VTE) [11.9% vs. 8.6%, *p* < 0.001], and depression [14.5% vs. 11.6%, *p* < 0.001]; and a lower prevalence of cancer [7% vs. 12%, *p* < 0.001], tobacco use disorder [6.9% vs. 12.2%, *p* < 0.001], transient ischemic attack (TIA) [6% vs. 7.5%, *p* < 0.001], and alcohol abuse [2.5% vs. 4%, *p* < 0.001] in the MHO cohort (Table 1).

The odds of dysrhythmia were higher in the MHO cohort. However, there was an obesity paradox with regard to MACCEs (OR 0.70, 95%CI 0.61–0.81, *p* < 0.001), all-cause mortality (ACM) (OR 0.66, 95%CI 0.54–0.82, *p* < 0.001), and acute myocardial infarction (AMI) (OR 0.71, 95%CI 0.59–0.86, *p* = 0.001) (Table 2).

## 4. Discussion

The rising incidence and prevalence of HFpEF correlate with increasing age and the prevalence of obesity, sedentariness, and cardiometabolic disorders [7]. Obesity, a key risk factor, is a major contributor to HFpEF, regardless of metabolic health status [8]. There is not a universally accepted definition of what constitutes “metabolically healthy obesity”. Commonly used criteria include the absence of metabolic syndrome components such as hypertension, dyslipidemia, high fasting glucose, and insulin resistance [9]. In the Veradigm Cardiology Registry’s cross-sectional analysis, a lower median age was noted among HFpEF patients with obesity, suggesting a possible earlier onset of HFpEF or higher mortality in older individuals with obesity, which could skew the age distribution [10]. In contrast, our study uniquely focuses on older adults who are HFpEF patients with metabolically healthy obesity, assessing MACCEs in this specific demographic. To our knowledge, this is the first retrospective study to assess major adverse cardiovascular and cerebrovascular events in older adults who are HFpEF patients with metabolically healthy obesity, compared to their metabolically healthy non-obese counterparts.

In this study, we observed that while MHO patients exhibited higher odds of dysrhythmia, they surprisingly showed reduced risks of MACCEs, ACM, and AMI compared to their non-obese (MHNO) counterparts. This “obesity paradox” suggests a complex interaction between MHO and health outcomes in HFpEF, with no significant differences in cardiac arrest (CA) and stroke rates between the groups.

### 4.1. Baseline Characteristics

The average age in our study group was 75 years in both the groups. Older adults with obesity are more likely to transition to a metabolically unhealthy state due to age-related metabolic changes, such as increased insulin resistance and inflammation. However, those who maintain a metabolically healthy status despite obesity might still face the mechanical burden of excess body weight on heart function, which can strain the heart over time. The prevalence of female sex was arguably higher, comprising 66% of the older adult HFpEF-MHO cohort. Women, especially pre-menopausal women, are more likely to exhibit the MHO phenotype compared to men. This is partly due to the protective effects of estrogen, which helps in maintaining insulin sensitivity and favorably influencing fat distribution (more subcutaneous fat compared to visceral fat). After menopause, the risk of transitioning from MHO to metabolically unhealthy increases in women. Comparatively, the reasons could be due to the fact that central obesity is even more common than general obesity in HFpEF, and there appear to be important sexual dimorphisms in its relationships with metabolic abnormalities and hemodynamic perturbations, with greater impact in women [11]. Obesity had a more deleterious effect on exercise capacity and diastolic function in women than in men, even in a healthy cohort. These subclinical changes might contribute to the development of a female predominance among HFpEF patients, particularly among individuals with obesity [12].

Baseline co-morbidities of our study, like COPD, hypothyroidism, prior VTE, and depression, were more prevalent in the MHO group. A retrospective cohort study, conducted using the OptumLabs Data Warehouse of U.S. administrative claims from 2008 to 2018, found that 50% of the patients with both HFpEF and COPD were also diagnosed with obesity. This suggests that obesity is a prevalent comorbidity in patients suffering from both conditions [13]. Hypothyroidism is notably prevalent among HF patients and aligns with our observations. In animal HF models, thyroid hormone supplementation enhanced diastolic function [14]. Moreover, treating clinical or subclinical hypothyroid older adult MHO-HFpEF patients with thyroid hormones could potentially improve not only cardiac symptoms but also extra-cardiac conditions such as reduced adiposity and better endothelial, arterial, and muscle functions. To date, randomized clinical trials have shown benefits primarily in HFrEF patients, as discussed by Razvi et al. [15], with no supporting preclinical or clinical studies on HFpEF patients. The ARIC (Atherosclerosis Risk In Communities) large prospective population-based study revealed a significant increase in VTE risk in patients hospitalized with HF. Notably, 27.7% of these patients had HFpEF, who were predominantly women with an average BMI of 30.2 and median age of 64.5. This study suggests a nearly fivefold increase in long-term VTE risk for HFpEF hospitalizations, highlighting an unexplored area since HFpEF patients have always been excluded from VTE risk assessments in heart failure randomized trials [16]. Our study mirrors the ARIC study’s demographics and also reports increased VTE prevalence in the MHO group, suggesting the need for further research into the mechanisms of VTE in this population and the impact of metabolic syndrome on outcomes.

### 4.2. Main Findings

#### Increased Risk of Dysrhythmias

The increased risk of dysrhythmia in older adult patients with MHO who have HFpEF can be attributed to several interconnected factors based on the current understanding of the pathophysiology of HFpEF and the impact of obesity, even when metabolic factors are not overtly detrimental. HFpEF and atrial fibrillation (AF) share similar risk factors like obesity, aging, and sleep apnea, which can also be the reason for increased dysrhythmias in this group [17]. The 32% increased risk of dysrhythmias observed in our MHO-HFpEF cohort is consistent with findings from a Korean study by Lee et al., which reported a 30% higher risk of arrhythmias in the MHO group compared to MHNO individuals [18]. The underlying pathophysiology is likely related to atrial arrhythmogenic remodeling, a central mechanism in the development of AF. Obesity promotes the progression of atrial fibrosis, which serves as a substrate for AF, and may be influenced by aging, systemic inflammation, or AF itself [19]. This aligns with observations in our study population of older adult patients with HFpEF and MHO, who, despite the absence of traditional cardiovascular comorbidities, may still demonstrate structural and electrical remodeling of the left atrium. Prior studies have shown that individuals with metabolically healthy obesity can exhibit left atrial enlargement, slowed conduction from the left atrium into the pulmonary veins, and a significantly shortened effective refractory period in both regions [20]. These alterations may increase susceptibility to atrial fibrillation and support the need for targeted rhythm surveillance in this phenotype.

MHO, along with HFpEF, has the following mechanisms:

1. Increases cardiac workload and blood volume, which stresses the heart and alters electrical activity, raising arrhythmia risks such as AF [21];
2. Structural changes like left ventricular hypertrophy (LVH) due to excess visceral fat disrupting [22] normal cardiac conduction;
3. Obesity-related autonomic imbalance enhances sympathetic and reduces parasympathetic activity, further predisposing to arrhythmias;
4. Increased volume of epicardial adipose tissue in obesity secretes pro-inflammatory and pro-fibrotic adipocytokines, which impair electrophysiological function and create a pro-arrhythmic substrate, increasing susceptibility to arrhythmias [22].

A prospective cohort study from Taiwan on the relationship between different obesity phenotypes and atrial fibrillation found that obesity, regardless of metabolic components, is associated with increased risk of AF [23]. One of the most unexpected and noteworthy findings in our study was that MHO patients had 32% higher odds of dysrhythmia compared to their MHNO counterparts. The increased dysrhythmia risk in MHO patients likely reflects obesity-related structural and electrical remodeling. These include atrial enlargement, epicardial adiposity, autonomic imbalance, and low-grade inflammation [24]. This can predispose to atrial fibrillation even in the absence of overt metabolic dysfunction. This finding highlights the need to explore therapeutic strategies that go beyond traditional risk factor modification. Recent evidence supports a potential arrhythmia-modifying role for GLP-1 receptor agonists, especially semaglutide. A meta-analysis of 26 randomized controlled trials involving nearly 49,000 participants found that semaglutide was associated with a 17% reduction in new-onset atrial fibrillation, independent of BMI, age, or glycemic status, with the greatest benefit seen when not combined with SGLT2 inhibitors [25]. This suggests GLP-1 receptor agonists may be particularly valuable in MHO individuals, whose arrhythmogenic risk may be driven more by obesity-related inflammation and fat distribution than by traditional metabolic pathways. In contrast, SGLT2 inhibitors, while beneficial in reducing arrhythmic events in HFrEF, have not demonstrated the same effect in HFpEF or HFmrEF [26]. Nevertheless, SGLT2 inhibitors are now the standard of care for HFpEF, based on evidence from the EMPEROR-Preserved [27] and DELIVER [28] trials, which showed up to a 20% reduction in HF hospitalizations and cardiovascular death, regardless of obesity status. Other novel agents also show promise. Finerenone, a mineralocorticoid receptor antagonist, is FDA-approved for patients with chronic kidney disease and type 2 diabetes and has demonstrated benefit in those with preserved ejection fraction [29]. Moreover, the SUMMIT trial [30] recently showed that tirzepatide, a dual GLP-1/GIP receptor agonist, significantly reduced the risk of cardiovascular death or HF worsening and improved quality of life in patients with HFpEF and obesity. Though not yet FDA-approved for HFpEF, tirzepatide may represent a valuable future option, especially for both MHO and MHNO populations. While our dataset (NIS 2019) offers robust national-level data, it predates the widespread use of sodium-glucose cotransporter-2 (SGLT2) inhibitors and glucagon-like peptide-1 (GLP-1) receptor agonists in HFpEF and lacks medication-level data. It is a well known recognized limitation of the NIS database.

### 4.3. The Paradox

The concept of the obesity paradox, particularly in HFpEF patients with obesity, suggests that factors such as excess energy reserves, a younger patient demographic, higher tolerability of HF therapy, and better nutritional status might contribute to improved survival outcomes [31]. In our study focusing on older adult MHO-HFpEF patients, we found lower odds of in-hospital MACCEs, all-cause mortality, and AMI when compared to MHNO-HFpEF patients.

Multiple studies, including the I-PRESERVE randomized controlled trial [32], research by Ather et al. [33], and others [34,35,36], have identified an obesity paradox in HFpEF patients, indicating a lower mortality risk compared to expectations. This finding contrasts with a 2019 meta-analysis of 43 studies [37], which reported higher risks for cardiovascular disease and all-cause mortality in MHO individuals, irrespective of their HFpEF status, compared to individuals with a metabolically healthy normal weight. This discrepancy underscores the need for further research to understand how metabolic health impacts outcomes across different HFpEF populations.

Several mechanisms aim to explain the obesity paradox in HF, with one suggesting that adiponectin, an adipocyte-specific cytokine inversely associated with BMI, may play a role [38]. Other proposed factors include altered pharmacokinetics in patients with obesity, which may enhance the tolerability of higher medication dosages that often pose a risk in patients without obesity due to hypotension. Additionally, obesity may lead to earlier detection and treatment of HF due to symptom mimicry, potentially improving outcomes [31].

The obesity paradox, where HFpEF patients with obesity appear to have better outcomes than patients without obesity, may be partly explained by factors not captured in our analysis. Residual confounding from unmeasured variables such as inflammation, physical fitness, or socioeconomic status, reverse causation where sicker patients lose weight and appear non-obese, and selection bias where patients with obesity may be admitted to the hospital more easily due to care complexity rather than illness severity, could all influence the results [39]. These explanations suggest that the observed survival benefit in patients with obesity may not be directly due to obesity itself, but rather other underlying differences between patient groups.

## 5. Limitations

This study is the first-ever large-scale nationwide analysis evaluating in-hospital outcomes among older adult patients with HFpEF stratified by metabolically healthy obesity status. We have also performed 1:1 propensity score matching to effectively balance key demographic and hospital-level characteristics between the MHO and MHNO cohorts. This is an observational analysis of administrative data. As a result, there is an inherent risk of residual confounding due to unmeasured variables, including physical activity, Frailty, diet, socioeconomic status, and medication adherence. This study used a 2019 sample, and as a result, the currently used SGLT2 inhibitors and GLP-1 receptor agonists were not FDA-approved in 2019. One of the other significant limitations of the NIS database includes the complete absence of medication data. Moreover, the NIS database does not include pharmacotherapy data, such as SGLT-2 inhibitors, GLP-1 receptor agonists, statins, beta blockers, ACE inhibitors, or ARBs. Additionally, the definition of metabolically healthy obesity was based solely on the absence of ICD10-CM codes for hypertension, diabetes, and hyperlipidemia, and this approach may result in misclassification, particularly in patients with subclinical disease or under-reported diagnoses. The Reliance on ICD-10-CM codes also introduces the risk of misclassification bias for both exposures and outcomes. Furthermore, the possibility of reverse causality must be acknowledged, as some images and no patients may have experienced unintentional weight loss due to underlying illnesses, which could confound comparisons. Another commonly encountered limitation is the difficulty in longitudinal follow-up, which restricts our ability to examine long-term complications, outcomes, or changes in the status of metabolically healthy obesity to unhealthy over time. Although we included propensity score matching, unmeasured confounders may still impact the validity of causal inferences.

## 6. Conclusions

Among individuals classified as metabolically healthy, obesity may still confer an elevated risk of dysrhythmia when compared to their non-obese counterparts. Despite the exclusion of traditional risk factors such as hypertension, diabetes, and hyperlipidemia, obesity appears to remain an independent contributor to increased dysrhythmia risk in elderly HFpEF phenotypes. Given the known potential for individuals with MHO to transition to metabolically unhealthy states over time, these findings suggest that discussing weight management strategies may be an important component of HFpEF care.

## Figures and Tables

**Table 1:** Baseline characteristics of HFpEF older adults with vs. without MHO status.

Variables		Metabolically Healthy Obesity (MHO)				*p* Value
		No		Yes		
		n	Percentage	n	Percentage	
Demographics						
**Total**		22,405		22,405		
**Age**	Median	75		75		
**Sex**	Male	7475	33.4%	7250	32.4%	
	Female	14,930	66.6%	15,155	67.6%	0.024
**Ethnicity**	White	18,230	81.4%	18,330	81.8%	0.018
	Black	2545	11.4%	2575	11.5%	
	Hispanic	925	4.1%	890	4.0%	
	Asian/Pacific Islanders	170	0.8%	130	0.6%	
	Native Americans	65	0.3%	80	0.4%	
	Others	470	2.1%	400	1.8%	
**Median household income national quartile for patient ZIP Code**	0–25th	6420	28.7%	6450	28.8%	0.292
	26–50th	5870	26.2%	6020	26.9%	
	51–75th	6000	26.8%	5920	26.4%	
	75–100th	4115	18.4%	4015	17.9%	
**Primary expected payer**	Medicare	20,200	90.2%	20,285	90.5%	0.005
	Medicaid	260	1.2%	285	1.3%	
	Private including HMO	1615	7.2%	1470	6.6%	
	Self-pay	175	0.8%	170	0.8%	
	No charges	<11	0.0%	<11	0.0%	
	Others	155	0.7%	190	0.8%	
**Elective versus non-elective admission**	Non-elective	21,170	94.5%	21,190	94.6%	0.768
	Elective	1235	5.5%	1215	5.4%	
**Bed size of the hospital**	Small	5085	22.7%	5465	24.4%	<0.001
	Medium	6930	30.9%	6670	29.8%	
	Large	10,390	46.4%	10,270	45.8%	
**Location/teaching status of hospital**	Rural	2640	11.8%	2515	11.2%	0.157
	Urban non-teaching	4625	20.6%	4610	20.6%	
	Urban teaching	15,140	67.6%	15,280	68.2%	
**Region of hospital**	NE	5040	22.5%	4975	22.2%	0.011
	MW	5415	24.2%	5715	25.5%	
	S	8190	36.6%	7990	35.7%	
	W	3760	16.8%	3725	16.6%	
**Comorbidities**						
**Alcohol abuse**		905	4.0%	560	2.5%	<0.001
**Depression**		2590	11.6%	3255	14.5%	<0.001
**Peripheral vascular disease**		2335	10.4%	2155	9.6%	0.0005
**Prior MI**		1305	5.8%	1125	5.0%	<0.001
**Prior TIA**		1680	7.5%	1340	6.0%	<0.001
**Prior VTE**		1920	8.6%	2655	11.9%	<0.001
**Drug abuse**		435	1.9%	390	1.7%	0.114
**Cannabis use disorder**		65	0.3%	35	0.2%	0.003
**Tobacco use disorder**		2730	12.2%	1550	6.9%	<0.001
**Opioid-related disorders**		415	1.9%	470	2.1%	0.062
**Chronic pulmonary disease**		10,210	45.6%	11,555	51.6%	<0.001
**Hypothyroidism**		4240	18.9%	5185	23.1%	<0.001
**Other thyroid disorders**		360	1.6%	370	1.7%	0.709
**Bariatric Surgery Status**		250	1.1%	535	2.4%	<0.001
**Cancer**		2695	12.0%	1560	7.0%	<0.001
**Secondary Outcomes**						
**Disposition of patient**	Routine	8585	38.3%	7550	33.7%	<0.001
	Transfers to short-term hospital	525	2.3%	450	2.0%	
	Transfer others *	6360	28.4%	7675	34.3%	
	Home Healthcare (HHC)	5505	24.6%	5835	26.0%	
	AMA	115	0.5%	85	0.4%	
**Length of stay (days), median [IQR]**		4		5		
**Total charges (USD), median [IQR]**		37,580.00		40,377.00		

IQR—Inter-quartile range; HMO—Health maintenance organization; MI—Myocardial Infarction; TIA—Transient Ischemic stroke; VTE—Venous thromboembolism; AMA—Against medical advice. * Includes skilled nursing facility (SNF), intermediate care facility (ICF), and another type of facility. *p* < 0.05 was considered significant.

**Table 2:** Primary outcomes.

Outcomes	Adjusted OR	95% CI Lower Limit	95% CI Upper Limit	*p* Value
**MACCEs**	0.7	0.61	0.81	<0.001
**All-Cause Mortality**	0.66	0.54	0.82	<0.001
**AMI**	0.71	0.59	0.86	0.001
**Dysrhythmia**	1.32	1.21	1.43	<0.001
**Cardiac Arrest, including VF**	0.98	0.66	1.46	0.93
**Stroke**	0.76	0.53	1.08	0.123

In the multivariable logistic regression analyses, the following list of factors and covariates were adjusted: Age at admission, Sex, Ethnicity, Median household income, national quartile for patient ZIP Code, Primary expected payer, Elective versus non-elective admission, Bed size of hospital, Location/teaching status of hospital, Region of hospital, Alcohol abuse, Arthropathies, Peripheral vascular disease, Depression, Drug abuse, Cannabis Use Disorder, Tobacco Use Disorder, Opioid-related disorders, Chronic pulmonary disease, Hypothyroidism, Other thyroid disorders, Prior TIA/Stroke without Neurologic deficit, Prior VTE, Prior MI, Bariatric Surgery Status, and Cancer.

## Data Availability

The data were obtained from the National Inpatient Sample (NIS) database. The datasets utilized are available from the corresponding author upon request.

## Supplementary Materials

The following supporting information can be downloaded at: https://www.mdpi.com/article/10.3390/jcm14155495/s1, Table S1: ICD-10-CM codes used for Obesity.

## Abbreviations

MHO (metabolically healthy obesity); HF (heart failure); HFpEF (heart failure with preserved ejection fraction); MACCEs (major adverse cardiovascular and cerebrovascular events); AMI (acute myocardial infarction); ACM (all-cause mortality); AF (atrial fibrillation); NIS (National Inpatient Sample); HCUP (Healthcare Cost and Utilization Project); AHRQ (Agency for Healthcare Research and Quality); IRB (Institutional Review Board); ICD-10-CM (International Classification of Diseases, Tenth Revision, Clinical Modification); CCSR (Revised Clinical Classifications Software); COPD (chronic obstructive pulmonary disease); VTE (venous thromboembolism); TIA (transient ischemic attack); MHNO (metabolically healthy non-obese); CA (cardiac arrest); LVH (left ventricular hypertrophy); SGLT-2 (Sodium-Glucose Cotransporter-2); GLP-1 (Glucagon like peptide-1); HFrEF (heart failure with reduced ejection fraction).
