# Supplementary material for: Impact of Metabolically Healthy Obesity on Cardiovascular Outcomes in Older Adults with HFpEF: Insights from a Nationwide Sample
Source: J Clin Med. 2025 Aug 4;14(15):5495. doi: 10.3390/jcm14155495 (PMC12346940; doi:10.3390/jcm14155495)
Supplement: Supplementary file 1 [file jcm-14-05495-s001.zip › jcm-3767573-supplementary.pdf]

**Obesity**	
E6601	Morbid (severe) obesity due to excess calories
E6609	Other obesity due to excess calories
E661	Drug-induced obesity
E662	Morbid (severe) obesity with alveolar hypoventilation
E668	Other obesity
E669	Obesity, unspecified
O99210	Obesity complicating pregnancy, unspecified trimester
O99211	Obesity complicating pregnancy, first trimester
O99212	Obesity complicating pregnancy, second trimester
O99213	Obesity complicating pregnancy, third trimester
O99214	Obesity complicating childbirth
O99215	Obesity complicating the puerperium
R939	Diagnostic imaging inconclusive due to excess body fat of patient
Z6830	Body mass index [BMI] 30.0-30.9, adult
Z6831	Body mass index [BMI] 31.0-31.9, adult
Z6832	Body mass index [BMI] 32.0-32.9, adult
Z6833	Body mass index [BMI] 33.0-33.9, adult
Z6834	Body mass index [BMI] 34.0-34.9, adult
Z6835	Body mass index [BMI] 35.0-35.9, adult
Z6836	Body mass index [BMI] 36.0-36.9, adult
Z6837	Body mass index [BMI] 37.0-37.9, adult
Z6838	Body mass index [BMI] 38.0-38.9, adult
Z6839	Body mass index [BMI] 39.0-39.9, adult
Z6841	Body mass index [BMI] 40.0-44.9, adult
Z6842	Body mass index [BMI] 45.0-49.9, adult
Z6843	Body mass index [BMI] 50.0-59.9, adult
Z6844	Body mass index [BMI] 60.0-69.9, adult
Z6845	Body mass index [BMI] 70 or greater, adult
